# AndroDex: Android Dex Images of Obfuscated Malware

**DOI:** 10.1038/s41597-024-03027-3

**Published:** 2024-02-16

**Authors:** Sana Aurangzeb, Muhammad Aleem, Muhammad Taimoor Khan, George Loukas, Georgia Sakellari

**Affiliations:** 1 National University of Computing and Emerging Sciences (FAST-NUCES), Department of Computer Science, Islamabad, 44000 Pakistan; 2 Centre for Sustainable Cyber Security, School of Computing and Mathematical Sciences, University of Greenwich, London, UK

**Keywords:** Mathematics and computing, Scientific data, Computer science

## Abstract

With the emergence of technology and the usage of a large number of smart devices, cyber threats are increasing. Therefore, research studies have shifted their attention to detecting Android malware in recent years. As a result, a reliable and large-scale malware dataset is essential to build effective malware classifiers. In this paper, we have created AndroDex: an Android malware dataset containing a total of 24,746 samples that belong to more than 180 malware families. These samples are based on .dex images that truly reflect the characteristics of malware. To construct this dataset, we first downloaded the APKs of the malware, applied obfuscation techniques, and then converted them into images. We believe this dataset will significantly enhance a series of research studies, including Android malware detection and classification, and it will also boost deep learning classification efforts, among others. The main objective of creating images based on the Android dataset is to help other malware researchers better understand how malware works. Additionally, an important result of this study is that most malware nowadays employs obfuscation techniques to hide their malicious activities. However, malware images can overcome such issues. The main limitation of this dataset is that it contains images based on .dex files that are based on static analysis. However, dynamic analysis takes time, therefore, to overcome the issue of time and space this dataset can be used for the initial examination of any .apk files.

## Background & Summary

Android smartphone applications are continuously gaining popularity due to the extensive use of mobile applications^1^. These applications serve various purposes, such as calling, messaging, data exchange, sending emails for correspondence and social interaction, browsing websites, controlling IoT-related devices, health monitoring, location tracking using GPS, online transactions, shopping, and are prevalent in almost every aspect of our everyday lives^2^. Apart from their user-friendly interactive environment and flexible operating system (OS), these applications are freely available on the official app stores, including the Google Play Store (www.google.com), the Apple App Store(https://www.apple.com/store), the Microsoft Store (https://apps.microsoft.com/) and the Amazon Store (https://www.amazon.com/). However, with the increase of mobile applications and their distribution, malicious apps and their variants are designed to track and spy on users’ behavior and activities, posing a threat to users’ privacy, confidentiality, and integrity^3,4^. While users tend to trust applications downloaded from official stores, the reality is different. Cybercriminals have started developing malicious mobile apps that exploit vulnerabilities and compromise users’ privacy through malware obfuscation techniques^5^. *Malware* is a malicious piece of software aimed at damaging systems without user consent^6,7^ and *malware obfuscation* is a technique used to defend against antivirus detection by hiding the program in a way that becomes difficult to understand^8^. Malware obfuscation techniques such as adding dump-code^9^, reassignment of registers^10^, subroutine reordering^10,11^, instruction substitution^8^, code transposition, and code integration^11^ can be applied to different types of malware such as Encrypted malware, Oligomorphic, Polimorphic, and Metamorphic Malware^12,13^. *Encrypted* malware represents the first step in evading antivirus signature-based security systems^13^. In encrypted malware, a decryptor is attached to the malware, aiming to recover the file after execution using different keys^14^, making the encryption complex and hiding its signature. However, anti-virus software can detect such malware by recognizing decryptor patterns. To overcome the limitations of encrypted malware, cybercriminals came up with the technology of mutating decryptors known as *oligomorphic* malware. However, oligomorphic can only mutate a few types of decryptor variants and, therefore, can be detected by anti-viruses^13^. To overcome this shortcoming, *polymorphic* malware generates an unlimited number of decryptors using obfuscation techniques, making it difficult to detect.

**Fig. 1:**
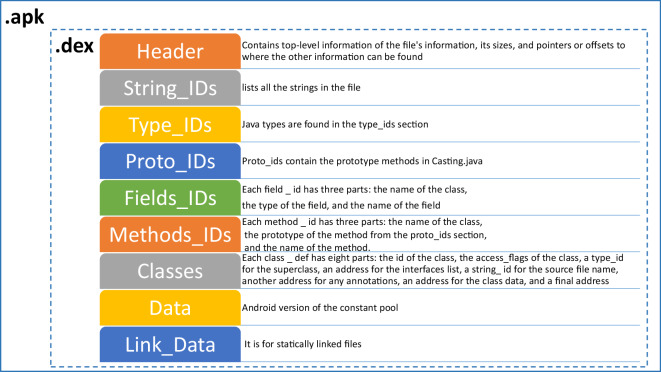
The Structure of DEX file^36^.

Android OS, released in 2008 and sponsored by Google, can run applications developed in Java. These codes are platform-dependent, which means they can only work if the target OS is Android. Android applications come up as an archive known as Android Package (APK)^15^. This APK file is a compressed package file usually in the format of .zip that comprises different libraries, directories, and records. This zip file consists of the Android Manifest file i.e., **AndroidManifext.xml**. This is a configuration file that contains meta-information about the application (i.e., the name of the application, the version number, permissions required, meanings of segments, for example, services, registration services, activities linked with other applications, content providers, broadcast receivers, libraries, and rendition support^2,16^. Then is the main and the most important **classes.dex** file, the runnable file on the Dalvik virtual machine, which contains all the operating instructions of the application and runtime data. The Android OS contains a folder named **res** that stores pictures, symbols, User Interface (UI) formats, and all the resource files needed by the APK. There is another folder named **libs** known as the library folder. Other library resources contain **assets** that store static files that need to be packaged into an APK. **META-INF** folder that stores application signatures and certificates to ensure the integrity of APK packages and system security, and lastly, the **resources.ars** file that is the compiled binary resource file as shown in Table 1. In this paper, we analyze classes.dex file structure as this is the only runnable file that contains all the operating instructions of the application and runtime data. Therefore, to analyze the application to be either as obfuscated or non-obfuscated .dex file plays a vital role. The structure of the .dex file is shown in Fig. 1.

**Table 1:** Structure of Android APK file.

Files and Folders	Description
Manifest (AndroidManifext.xml)	A required configuration file contains Key information about the application. For example, the application’s package name, its components i.e., activities, resources, permissions requires to run and to access this application’s information by other apps, compatibility features i.e., minimum Android version and supported devices^16^.
Delvik Bytecode (classes.dex)	The only runnable file on the Dalvik virtual machine, which contains all the operating instructions of the application and runtime data. APK files may contain more than one classes.dex file that will be numbered as classes2.dex, classes3.dex, and so forth^16^.
Resources (res/)	stores pictures, symbols, User Interface (UI) formats and all the resource files stored in the folder hierarchy required by the APK and to be used by the developer^16^
Native Libraries (libs/)	The library folder that contains native libraries (machine code)^16^
Assets (assets/)	store static files that need to be packaged into APK^16^.
Signatures (META-INF)	Folder that contains verification information and store application signatures and certificates to ensure the integrity of APK packages and system security. This means that any change in the APK file must require resigning the APK, otherwise, the OS will reject the installation^16^.
Compiled Resources (resources.arsc)	the compiled binary resource file that contains information that links the code (classes.dex) to the resources (res)^16^.

Keeping in mind the above discussion, as obfuscation techniques become more sophisticated, the future trend of classifying applications into malicious, benign, and obfuscated malware is increasing. In this dataset, we have converted .dex files of both benign and malware applications into images. Additionally, we have applied obfuscation techniques to demonstrate how images play a vital role in identifying obfuscated malware. To analyze critical malware apps i.e., the obfuscated malware, the existing datasets are Kronodroid, Drebin, Malgenome, and AndroZoo datasets. Kronodroid contains a vast range of malware from the year 2008 to 2020. However, Drebin, Malgenome, and AndroZoo datasets are mainly used for the classification of malware and benign applications and lack a good number of advanced malware such as those malware that employs several encryption techniques (e.g., polymorphic behavior). None of the existing datasets contains images of the obfuscated malware, whereas, with technological advancements, the malware are becoming more sophisticated and older malware samples are not adequate for the analysis of the newer malware threats based on obfuscation techniques. Additionally, the dataset size in terms of the number of samples are smaller as compared to the employed Androdex dataset. For example, the MalGenome dataset contains 1260 samples whereas, the Drebin dataset consists of 5560 samples from the year 2010 to 2012, kronodroid consists of 28,475. In contrast, the dataset employed in this study i.e., Androdex contains more diversified malware samples i.e., the older ones and with new and advanced samples along with the obfuscated ones (including Kronodroid) samples total 45,879 of which 24,746 are binaries and 21,133 are images. Furthermore, the Androdex dataset consists of images as well as binary format that provide dual flexibility to classify malware using supervised and unsupervised methods. In addition, images plays a vital role in detecting obfuscated malware, therefore, latest neural network algorithms can be applied to identify malware in an advanced way.

## Methods

### Dataset acquisitions

**Fig. 2:**
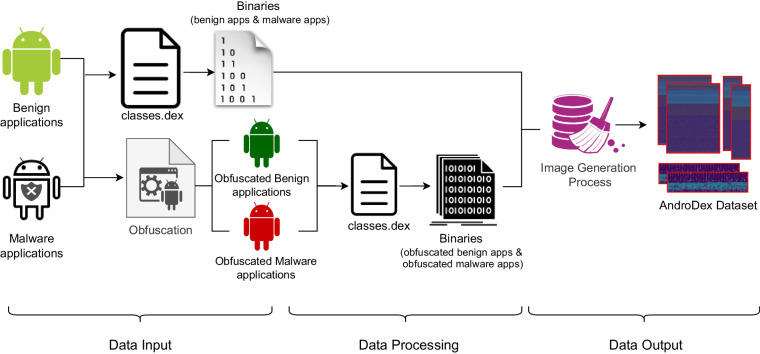
Workflow representing the data processing steps to obtain AndroDex Dataset.

**Fig. 3:**
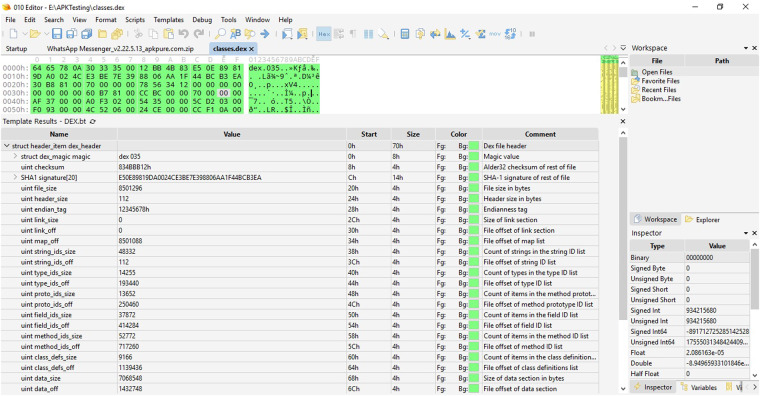
010 editor view of classes.dex files as decimal format.

**Fig. 4:**
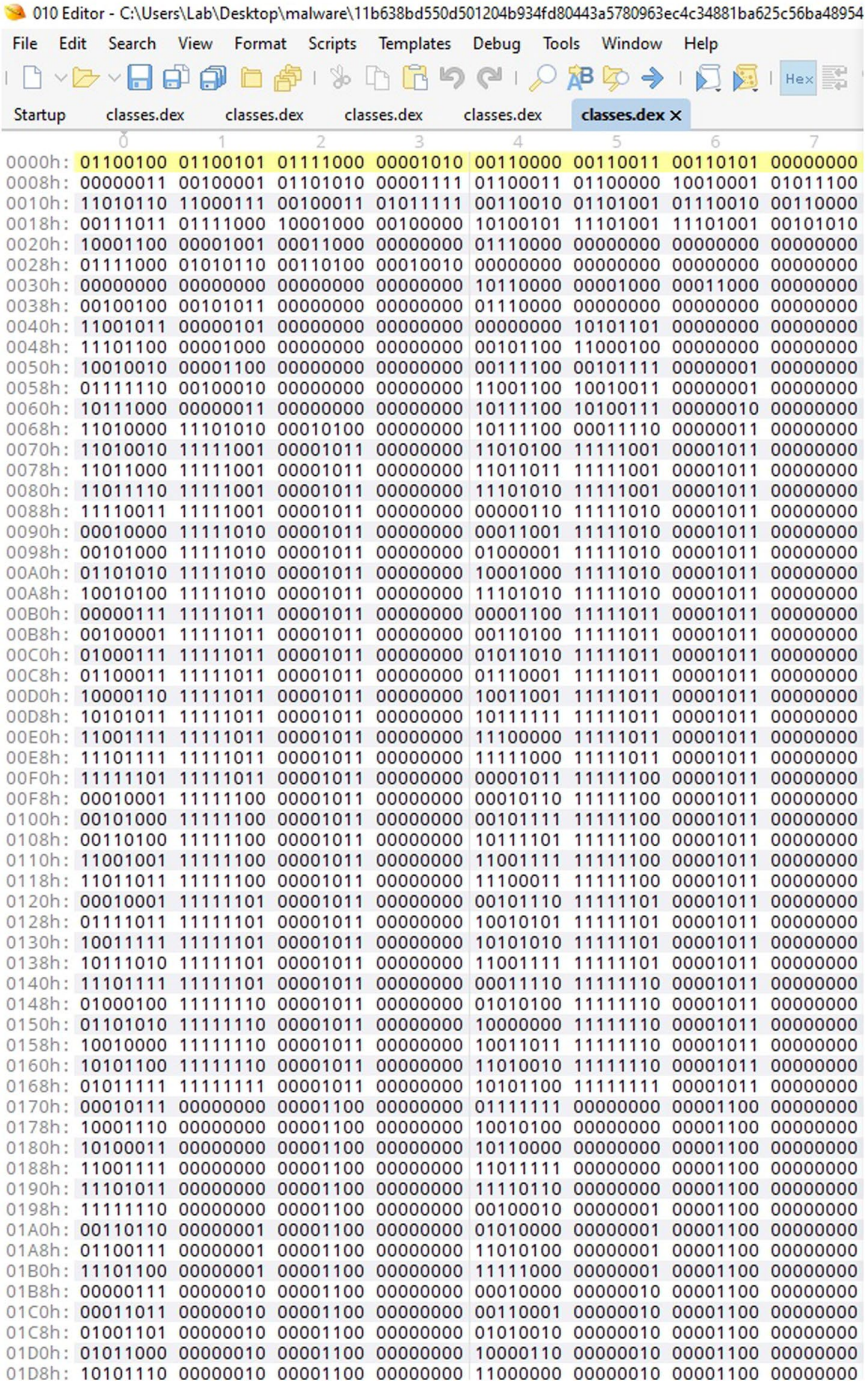
010 editor view of classes.dex files as binary format.

The AndroDex dataset^17,18^ consists of 24,746 binaries of which 21,133 images are successfully converted against android .dex file which consists of benign images, malware images, obfuscated-benign images, and obfuscated-malware images as shown in Table 2. To construct the dataset, we used application hash values from three well-known and widely used datasets (i) Drebin^19^ (ii) Kronodroid^20^ that covers a wide variety of malware (iii)Androzoo^21^. The Drebin dataset consists of 5,560 files from 179 different malware families whereas Kronodroid consists of 28,745 malicious apps from 209 malware families and 35,246 benign samples whereas Androzoo contains more than three million unique Android apps. Unfortunately, these datasets provide the hash values only, therefore, the first challenge is to get the APK files. Once the APK files are downloaded which is a compressed package file usually in the format of .zip. After extracting the .zip file we get the most important runnable file comprised of all the important operating instructions of the application i.e., classes.dex file as shown in Fig. 2. To construct the dataset, these classes.dex files are then converted into their respective binary files using the 010 editor. The 010 editor can provide both the decimal and binary format of .dex file as shown in Figs. 3, 4 respectively. So, we automate this step for all the datasets in order to get the binary files of the respective DEX files. To construct images, binary and decimal values are equivalent in such a way that binary values 0 represents the black color and 1 represents the white color and for grey-scale images, we used an 8-bit color format which is one of the most famous image formats. Therefore, behind the image matrix value ranges are from 0–255 where 0 represents black and 255 represents white. For colored images 16-bit format or 24-bit format is used in such a way that 16-bit format is further divided into Red, Green, and Blue (R,G,B) format.

**Table 2:** Dataset Detail.

Obfuscation Type	Dataset	Malware/Benign	Total Binaries	Total Images
AVPass	Kronodroid/AndroZoo	benign	838	836
AVPass	Kronodroid	malware	1588	1586
AVPass	Kronodroid	obfuscated malware	1600	1562
Original	Kronodroid/AndroZoo	benign	4745	3988
Original	Drebin	malware	5554	3576
Obfuscapk	Kronodroid/AndroZoo	obfuscated benign	4885	4300
Obfuscapk	Drebin	obfuscated malware	5536	5285

### Dataset overview

To construct the AndroDex dataset^17,18^, we relied first on two classes i.e., malware and benign applications (see Fig. 2). However, later we applied two types of obfuscation techniques (i) AVPass^22^ (ii) Obfuscapk^23^ to obfuscate the benign and malware applications that give us the count of 4 classes (i) Benign (ii) Malware (iii) Obfuscated Benign (iv) Obfuscated Malware (see Table 2). After applying obfuscation we generated the binaries of these files which are then converted into colored images. Summarizing the methodology is as follows:

- benign and malware applications’ APK files are gathered
- extract the .zip file of the respective APK to get the classes.dex file
- for each classes.dex files we have generated the binary files
- obfuscation is being applied on benign and malware applications to get the two more classes: obfuscated benign and obfuscated malware.
- the binary files are generated against the two new obfuscated classes from their classes.dex files
- binaries of all four classes are then converted into colored images

#### Obfuscation

Malware, a well-known term is a malicious piece of software, used by attackers with the aim of breaching the integrity, confidentiality, and authenticity of the computer system and user privacy^6^ whereas malware obfuscation is a technique of defending anti-viruses which means hiding the program in a way that becomes difficult to understand. Malware obfuscation techniques such as adding dump-code, reassignment of registers, subroutine reordering, instruction substitution, code transposition, and code integration can be applied to different types of malware such as Encrypted malware, Oligomorphic, Polymorphic, and Metamorphic Malware^12^. To construct AndroDex image-based dataset, we have employed two types of obfuscation techniques to generate two sets of images (i) AVPass^22^ and (ii) Obfuscapk^23^. The main aim is to identify the behavioral pattern of malware and benign applications through images. There exist many obfuscated benign applications that are actually not malicious but are obfuscated just to protect the data. However, because of obfuscation anti viruses usually label those classes as malware. Therefore, it becomes compulsory to identify legitimate obfuscated applications from malicious obfuscated applications through images. As images reflect the true representation of the applications, therefore, the AndroDex dataset can be used for the classification and identification of malware and benign applications.

Using AVPass^22^, we have applied six different types of obfuscation:

- API_Reflection
- String encryption
- Variable name encryption
- Package name encryption
- Method and Class name encryption
- Resource encryption

Using Obfuscapk^23^, we have applied four different types of obfuscation:

- Renaming: ClassRename, FieldRename, MethodRename
- Encryption: LibEncryption, ResStringEncryption, AssetEncryption, ConstStringEncryption
- Code: ArithmeticBranch, Reorder, CallIndirection, DebugRemoval, Goto, MethodOverload, Nop
- Reflection: Reflection, AndvancedReflection

### Image generation process

To construct the AndroDex dataset, we have used DEX files which consist of 8-bit binary. For images, pixels are used whereas a pixel itself is an 8-bit binary vector^24^. Therefore, the bytes of DEX files are converted to pixels that can effectively save time in extracting features from typical datasets. The DEX file consists of three main sections: Header Section, Index Section, and Data Section. We have considered all three sections to generate images that could be huge in size. Therefore, the rule for image generation is as follows:

If the DEX file size is < 10 kilobytes then the image matrix dimension would be 32 whereas if the DEX file size is equal to 10 kilobytes or less than 30 kilobytes then the image matrix dimension would be as 64. Similarly, if the DEX file size is equal to 30 kilobytes or less than 60 kilobytes the image matrix dimension would be 128, and in case the DEX file size is equal to 60 kilobytes or less than 100 kilobytes the image matrix dimension would be as 256. If the DEX file size is equal to 100 kilobytes or less than 200 kilobytes the image matrix would be 384. Moreover, in case the DEX file size is equal to 200 kilobytes or less than 500 kilobytes the image matrix dimension would be as 512. If the DEX file size is equal to 500 kilobytes or less than 1000 kilobytes the image matrix would be 768 and lastly, if the DEX file size is more than 1000 kilobytes then the image matrix would be as 1024 as shown in Table 3.

**Table 3:** Image Conversion Rule^37^.

DEX file size	Image Matrix
< 10 KB	32
10 KB–30 KB	64
30 KB–60 KB	128
60 KB–100 KB	256
100 KB–200 KB	384
200 KB–500 KB	512
500 KB–1000 KB	768
> 1000 KB	1024

To generate images in colored .jpg format, we have used Python libraries such as matplotlib.pyplot, numpy, os, math, and shut function, which take binaries file folder path as input, and generate images one by one. For detail code analysis see section Code Availability and image conversion code^18^.

## Data Records

AndroDex dataset^17,18^ consists of two main folders *Set1* and *Set2*. Set1 is based on the AVPass obfuscation techniques whereas Set2 is based on obfuscated techniques. Set1 consists of images belonging to three classes i.e., benign, malware, and obfuscated malware. Set2 consists of images from four classes: benign, malware, obfuscated benign, and obfuscated malware. The data records including the repository is available online (see section Code Availability^17,18^). In addition, the records contain a folder *AndroDex_code* with all the codes, script and intermediary data to reproduce the dataset or to add new indicators or new surveys. The folder *AndroDex_binaries* included all the binary files in .txt format so the researchers can use these files to generate images using their own parameters which will help them identify malware accordingly.

## Technical Validation

To validate the AndroDex dataset, we proceeded by evaluating the AndroDex using several machine-learning models for classifying malware. Since machine learning models can use images as input for the classification process and can attain high accuracy over several challenging problems such as object detection, object classification, and identification. Therefore, we developed an approach to evaluate by applying various machine learning models to the benchmark dataset for evaluation and comparison purposes. For this purpose, we have evaluated the images using different matrices such as 64 × 64, 128 × 128, and 256 × 256. The parameters used for evaluation are accuracy, precision, recall, and F1-score. The results of the classifiers without normalization are displayed in Table 4 whereas the results of all the classifiers after normalization are displayed in Table 5.

The training set images have been normalized before use and for simplicity, Principle Component Analysis (PCA) is applied to normalized data. Later, the normalized and reduced features are given as input to machine learning classifiers to test the data. To cross-validate the data K-fold cross-validation is used. Lastly, to evaluate the classifier’s performance metrics like accuracy, precision, recall, and F1-Score are used. The results of which can be seen in Table 5.

**Fig. 5:**
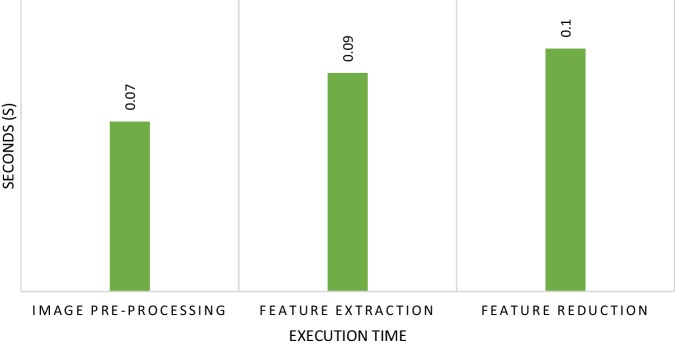
Execution time taken during image pre-processing, feature extraction and feature reduction.

The execution time taken by the proposed approach for pre-processing of the image is 0.07 s whereas it takes 0.09 s for feature extraction and 0.1 s for feature reduction against the total 21,133 images including both sets of data. For training we have used 80% of the images and 20% of the images are used for testing. The total training process took 2.5 s. The graphical representation can be seen in Fig. 5. However, the limitation of using this dataset is that images are based on .dex files that is statically analyzed whereas dynamic analysis takes a lot of time and space therefore, to overcome the issue of time and space this dataset can be used for the initial examination of any apk files.

### Machine learning algorithms

Several Machine Learning (ML) algorithms such as SVM, KNN, XGBoost, and RF are applied to analyze the malware images.

#### SVM

SVM is an ML model based on supervised learning that analyzes data for classification purposes. SVM learns from the past input data and makes future predictions as output using a mapping function s = f(x); where *s* is a scalar that represents one of the categories in order to build a model, and *x* is a high-dimensional feature vector containing numerical values^25^. For this purpose, the SVM needs a training set with each example labeled as belonging to one of two categories such as malware or benign in our case. Multi-class SVMs (MCSVM) can also be implemented by combining several binary SVMs^26^. SVM uses hyperplanes that have the maximum distance to the support vectors of any class to create a classifier with a maximal margin. The aim of SVM is to find the largest distance margin that leads to getting the optimal hyperplane to produce good results. The chances of misclassification increase if the hyperplane has a low or no margin. If the classes cannot be separated linearly, SVM can turn this space into a higher-dimensional feature space. Kernel functions, such as polynomials, radial basis functions (RBF), or sigmoid functions, are used to accomplish this. We have used linear, RBF, polynomials, and sigmoid for classifying obfuscated malware images.

#### KNN

K-Nearest Neighbors also termed as KNN is a supervised ML classification algorithm. It is a non-parametric classification method which is a simple yet effective method for classification^27^. For a data record *d* to be classified, its *k* nearest neighbors are redeemed, and thus creates a neighborhood of *d*. It is a method to classify data *d* based on the closest samples from a neighbor. However, the unknown data points are classified by majority votes from chosen *k*. To apply kNN, we select an acceptable value for *k* with uniform weights for predictions, and thus the classification’s outcome heavily depends on this *k* value. In another way, the kNN method is biased by *k*. There are numerous methods for selecting the *k* value, but one straightforward one is to repeatedly run the algorithm with various *k* values and select the one that performs the best.

#### Random forest

One of the most well-known and effective ensemble-supervised machine learning techniques is the Random Forest (RF) algorithm by Leo Breiman^28^. An approach known as an ensemble learner of classification trees^29^ produces numerous individual learners and aggregates the outcomes. RF uses the bagging method^30^ for improvement, where, each classifier is constructed separately by working with a bootstrap sample of the input data. In a typical decision tree classifier, a decision is taken based on all of the feature properties at a node split, however, in RF, the best parameter at each node in a decision tree is made from a randomly selected number of features^31^. This random feature selection lessens the interdependence (correlation) between the feature properties. Thus, this approach is less susceptible to the data’s intrinsic noise^32^.

For validation, we use an RF classifier which is formed by a bunch of decision trees^33^. And we use the Python ski earn library function: srf = RF(n _ estimators = n, njobs = −1) to build the random forest with several trees number. And use the python sklearn library function: srf.fit(x _train, Ltrain) to input the training datasets and use the python sklearn library function: srf.score(x _test, y _test) to see the result shown in Table 4 without normalization and Table 5 after normalization.

**Table 4:** Classifiers and their Accuracy, Precision, Recall and F1-score without Normalization.

Classifier	Image Matrix	Accuracy	Precision	Recall	F1-Score
SVM	64	86%	0.80	0.88	0.84
	128	86%	0.83	0.83	0.83
	256	88%	0.83	0.89	0.86
KNN	64	80%	0.72	0.87	0.79
	128	81%	0.78	0.76	0.77
	256	83%	0.85	0.72	0.78
RF	64	83%	0.75	0.89	0.82
	128	84%	0.76	0.86	0.81
	256	84%	0.79	0.85	0.80
XGBoost	64	86%	0.80	0.88	0.84
	128	86%	0.83	0.83	0.83
	256	88%	0.83	0.89	0.86

**Table 5:** Classifiers and their Accuracy, Precision, Recall and F1-score after Normalization.

Classifier	Image Matrix	Accuracy	Precision	Recall	F1-Score
SVM	64	88%	0.82	0.88	0.85
	128	89%	0.84	0.85	0.85
	256	90%	0.88	0.89	0.89
KNN	64	82%	0.73	0.87	0.79
	128	82%	0.77	0.77	0.77
	256	84%	0.86	0.74	0.79
RF	64	90%	0.88	0.90	0.90
	128	91%	0.89	0.89	0.90
	256	94%	0.90	0.90	0.91
XGBoost	64	92%	0.88	0.88	0.89
	128	94%	0.91	0.92	0.92
	256	95%	0.90	0.92	0.92

#### XGBoost

XGboost stands for eXtreme Gradient Boosting package is a supervised algorithm built on ensemble trees and an extension of gradient boosting. It is an efficient, prominent, and scalable classifier for the implementation of a gradient-boosting framework. In addition, it achieves good performance as it has several features such as speed, high expandability, input type, sparsity, customization, and performance^34,35^. The package comprises of optimized linear model solver and tree learning algorithm with regularization term and loss function. It supports Generalized Linear Machine Learning algorithms and GBDT model to implement in Gradient Boosting Framework. The basic model of GBDT are Regression Tree or CART (classification and regression tree)^35^. For XGBoost we gave used the default parameters.

## Usage Notes

The AndroDex dataset is provided in binary as well as image format so it can be easily used in any data processing software. The images and binaries can be easily opened and processed using Notepad, R, python, WEKA, or any other software whereas .txt files can be used and opened in any format. These files can be easily converted into .csv format to ready by Python, R, WEKA, etc. All the files are password protected and the password is *androdex*. The files are archived and password protected, however, the password is publicly availabe to re use this dataset. The reason of using password is to make sure that none of the files were mistakenly deleted by server by considering them malicious. As the files are malicious so server usually delete them, therefore for safety purpose the password is enabled. User can download the folder, extract the files by entering *androdex* password and use all the images easily.

## Data Availability

The dataset *AndroDex* is publicly available and can be accessed via the following links: Binaries of all files along with the code to convert images of any size are available at^18^: 10.6084/m9.figshare.23931477.v1, whereas images converted are available at^17^ 10.6084/m9.figshare.23931204.v1. All the files are password protected to make sure that none of the files were deleted by server and the password is *androdex*. The *AVPass obfuscation technique* that applied is available at (https://github.com/sslab-gatech/avpass) whereas *Obfuscapk technique* is available at (https://github.com/ClaudiuGeorgiu/Obfuscapk).
