# Author Correction: AndroDex: Android Dex Images of Obfuscated Malware

**DOI:** 10.1038/s41597-024-03586-5

**Published:** 2024-07-04

**Authors:** Sana Aurangzeb, Muhammad Aleem, Muhammad Taimoor Khan, George Loukas, Georgia Sakellari

**Affiliations:** 1 National University of Computing and Emerging Sciences (FAST-NUCES), Department of Computer Science, Islamabad, 44000 Pakistan; 2 Centre for Sustainable Cyber Security, School of Computing and Mathematical Sciences, University of Greenwich, London, UK

**Keywords:** Mathematics and computing, Scientific data, Computer science, Mathematics and computing, Scientific data, Computer science

Correction to: *Scientific Data* 10.1038/s41597-024-03027-3, published online 16 February 2024

In this article the reference “Nataraj, L. *et al*. Malware images: visualization and automatic classification. *VizSec '11: Proceedings of the 8th International Symposium on Visualization for Cyber Security*. 10.1145/2016904.2016908 (2011)” was missing and should have been cited in the Table 3 caption as ‘Table 3. Image Conversion Rule^37^’. The original article has been updated.

